# Supplementary material for: Investigation of hemodynamic bulk flow patterns caused by aortic stenosis using a combined 4D Flow MRI-CFD framework
Source: PLoS Comput Biol. 2025 Mar 27;21(3):e1012467. doi: 10.1371/journal.pcbi.1012467 (PMC11996075; doi:10.1371/journal.pcbi.1012467)
Supplement: S1 Table — (PDF) [file pcbi.1012467.s001.pdf]

	**Healthy**	**Aortic valve stenosis**
**Image orientation**	Axial	Sagittal
**Spatial resolution**	1.563 mm x 1.563 mm x 1.56 mm	0.789 mm x 0.789 mm x 1.4 mm
**Temporal resolution**	24.2 ms	34.4 ms
**Velocity encoding**	150 cm/s	120 cm/s
