# Supplementary material for: Investigation of hemodynamic bulk flow patterns caused by aortic stenosis using a combined 4D Flow MRI-CFD framework
Source: PLoS Comput Biol. 2025 Mar 27;21(3):e1012467. doi: 10.1371/journal.pcbi.1012467 (PMC11996075; doi:10.1371/journal.pcbi.1012467)
Supplement: S1 Methods — (PDF) [file pcbi.1012467.s002.pdf]

S1 Method. Mesh independency study and mesh parameters.

ANSYS ICEM CFD (ANSYS, Inc., Canonsburg, PA) was used for mesh generation. Here, an unstructured grid with tetrahedral elements was applied for the bulk region, and the near-wall region contained layers of prism elements. A mesh independence study was carried out for three parameters of interest and three subsequent steps using the aortic valve stenosis (AS-78) model, since the AS flow was expected to be more complex, thus posing higher requirements for mesh independency than the healthy (H-25, H-78) flow. Therefore, it was assumed that mesh independency acquired for the AS model would also be suitable for the healthy case. According to Celik et al. [1], the grid refinement factor R for grid size h should be at least 1.3 between subsequent meshes to yield significant changes:

$$R = \frac{h_{coarse}}{h_{fine}} \geq 1.3 \tag{1}$$

First, the prism elements in wall vicinity were defined in relation to the resulting maximum Y+ value in the entire domain. The height of the initial layer was adjusted gradually until Y+ dropped and remained below 1. This was achieved for an initial height of 0.0125 mm.

Next, the tetrahedral elements were investigated. Due to significantly differing flow conditions in the ascending and the descending aorta, the requirements for a suitable mesh were expected to differ accordingly. Therefore, the mesh independency was obtained for each region separately. For the aortic arch and the descending aorta, the entire aortic model was utilized for the mesh independency analysis. For the ascending aorta, however, since significantly smaller element sizes were expected to be required, the model was cut at the aortic arch in order to reduce the total amount of elements. This enabled computationally less expensive calculations. The interface at the modified outlet was defined by using pressure values extracted from the corresponding simulation results of the entire aorta.

For both regions, the maximum element size was reduced gradually until the systolic, area-averaged values for the velocity and the total shear stress on cross-sectional ROIs along the ascending or descending aorta, as well as the area-averaged wall shear stress on the entire wall domain converged with a percentage change between two subsequent meshes below 5 %.

The final mesh setting parameters are presented in Table A in S1 Method and Fig A in S1 Method.

Table A in S1 Method Mesh setting parameters obtained after mesh independency analysis

**General mesh information**		
Number of nodes	1.23 million	
Number of elements	4.65 million	
**Prism elements**		
Initial height in mm	0.0125	
Height ratio	1.2	
Number of layers	12	
**Tetrahedral elements**	**Ascending aorta**	**Aortic arch and descending aorta**
Scale Factor	1	1
Max element	0.5	2
Curvature min size limit	0.5	0.5
Elements in gap	1	1
Refinement	30	30

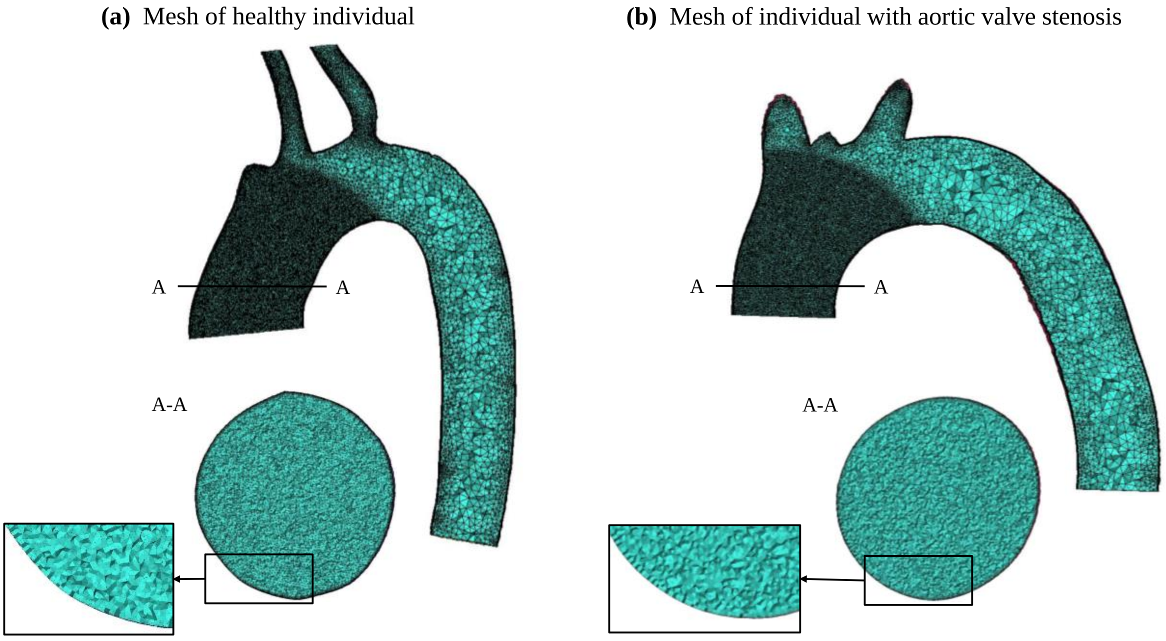

Fig A in S1 Method Meshes shown on a sagittal and cross-sectional cut plane, including a zoomed in view of the boundary layer. (a) Healthy individual H-25, (b) Individual with aortic stenosis AS-78, also used for H-78

References

1. Celik I, Ghia U, Roache PJ, Freitas C, Coloman H, Raad P. Procedure for Estimation and Reporting of Uncertainty Due to Discretization in CFD Applications. J. Fluids Eng. 2008;130:78001. doi:10.1115/1.2960953.
