# Supplementary material for: Investigation of hemodynamic bulk flow patterns caused by aortic stenosis using a combined 4D Flow MRI-CFD framework
Source: PLoS Comput Biol. 2025 Mar 27;21(3):e1012467. doi: 10.1371/journal.pcbi.1012467 (PMC11996075; doi:10.1371/journal.pcbi.1012467)
Supplement: S2 Methods — (PDF) [file pcbi.1012467.s003.pdf]

S2 Method. Implementation of Loss Coefficient Model in Theory and ANSYS CFX.

In practice, the loss coefficient (LC) model involves an iterative process of continuously adjusting the LC values in every numerical timestep. In an inner coefficient loop, the following steps are being carried out iteratively for each outlet where the model is prescribed. The expressions for an implementation in ANSYS CFX are shown.

1. Measurement of current mass flow $mOUTLET$ through opening and difference $diffOUTLET$ to target percentage $perc$ of $INLET$ mass flow

$$mOUTLET = massFlow()@OUTLET \quad (1)$$

$$diffOUTLET = abs\left(\frac{mOUTLET}{perc * massFlow()@INLET}\right) - 1 \quad (2)$$

2. Definition of an additional variable transOUTLET which gets passed on and updated from timestep $t = n$ to timestep $t = n + 1$: $transOUTLET(t = n + 1) = transOUTLET(t = n) + difference$

$$transOUTLET = transOUTLET + diff(OUTLET) * 10 \quad (3)$$

   Remark: In ANSYS CFX, the source code to variable transOUTLET needs to be modified by adding the line Update Loop = TRANS LOOP. This enables the recurse to its value in the previous timestep.

3. The area averaged value of $transOUTLET$ quantifies the LC value at the outlet. Based on the underlying LC equation (see Equation (2) in the manuscript), the corresponding pressure value is calculated and prescribed as boundary condition to the 3D model.
