# Supplementary material for: Investigation of hemodynamic bulk flow patterns caused by aortic stenosis using a combined 4D Flow MRI-CFD framework
Source: PLoS Comput Biol. 2025 Mar 27;21(3):e1012467. doi: 10.1371/journal.pcbi.1012467 (PMC11996075; doi:10.1371/journal.pcbi.1012467)
Supplement: S3 Methods — (PDF) [file pcbi.1012467.s004.pdf]

S3 Method. Generation of synthetic healthy inflow profile based on pathological in-vivo 4D Flow MRI measurements.

The goal in defining the synthetic healthy inflow profile is to generate a physiological flow profile for a pathological case (patient suffering from a cardiovascular disease, here AS) while matching the cardiac output of that patient. Thus, the volume flow at each timestep of the cardiac cycle, where in-vivo data of the patient are available, is treated as the target volume flow the synthetic inlet flow of the synthetic profile has to meet. In the given cases, this results in 20 discrete measurement points, corresponding to the 20 MRI acquisitions over one cardiac cycle, for the AS-78 patient. These 20 discrete inflow profiles are then interpolated to generate a continuous inlet velocity profile over time.

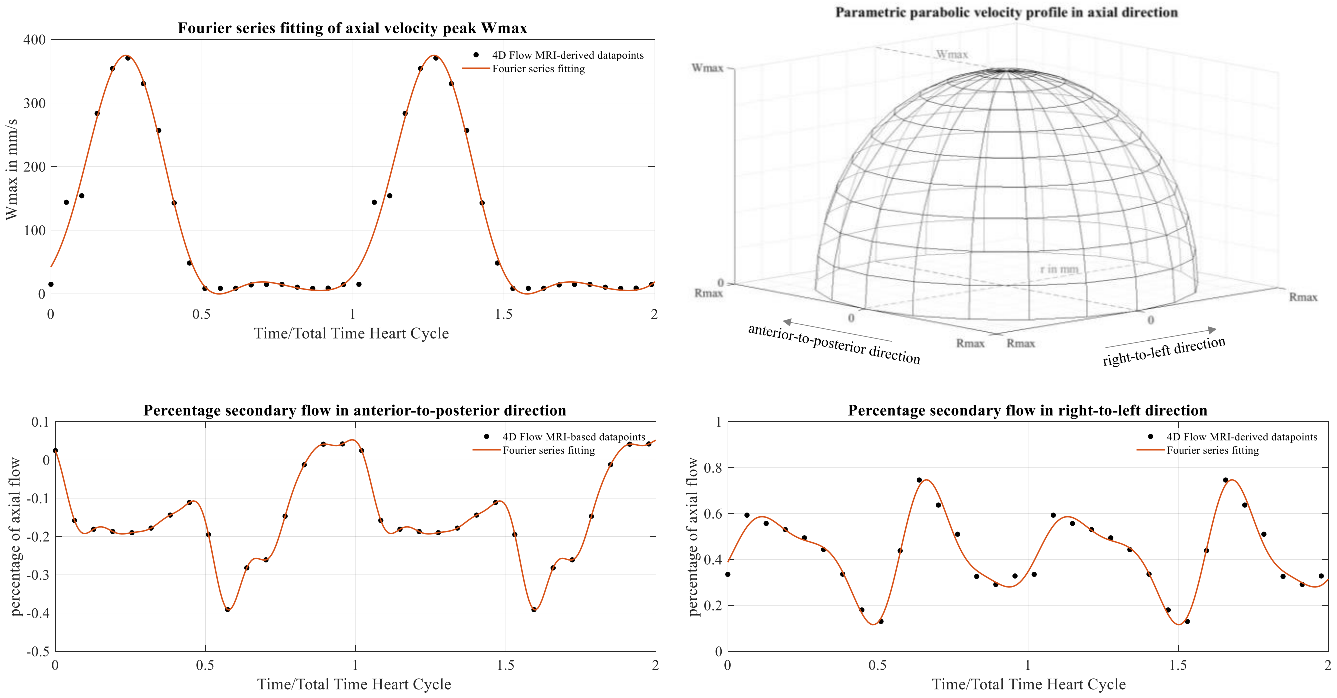

Fig A in S3 Method Fourier series fittings of the three dimensional synthetic flow with parabolic profile. Upper right: Parametric drawing of the parabolic profile. Upper left: axial velocity over time fitted to match the AS-78 volume flows, see Equation (3). Bottom: Percentages of secondary flow fitted to match the H-25 secondary flow percentages, see Equation (6) for the right graph, Equation (7) for the left graph. Black dots represent the timepoints where 4D Flow MRI reference measurements are available.

The synthetic healthy inflow is based upon a parabolic profile with radius r = R_{max}, as shown in Fig A in S3 Method. The black dots represent the MRI timesteps. It defines the axial velocity W, that changes its momentary maximum value $W_{max}(t)$ over the course of the cardiac cycle, see Equation (1).

$$W = W_{max}(t) \cdot (1 - \frac{r}{R_{max}})^{1/7}, with\, r = \sqrt{x^2 + y^2} \tag{1}$$

The volume flow $\dot{V}$ introduced by the parabolic inflow equals to the volume of the paraboloid and is calculated by Equation (2).

$$\dot{V} = \frac{4}{3}\pi \cdot W_{max}(t) \cdot r^2 \quad \Leftrightarrow \quad W_{max}(t) = \frac{4}{3}\frac{\dot{V}}{\pi r^2} \tag{2}$$

For each MRI timestep, the value of W_{max} is adjusted in order to generate a volume flux that is equal to the corresponding AS-78 case at that timestep. The resulting discrete W_{max} function is then fitted to a third-degree Fourier series, shown in Equation (3).

$$\begin{aligned} W_{max}(t) = \; & a0 + a1 \cdot cos(tw) + b1 \cdot sin(tw) + a2 \cdot cos(2tw) + b2 \cdot sin(2tw) \\ & + a3 \cdot cos(3tw) + b3 \cdot sin(3tw) \end{aligned} \tag{3}$$

With the coefficients a0 = 110.6 mm/s, a1 = 32.8 mm/s, b1 = 159.1 mm/s, a2 = -78.67 mm/s, b2 = 31.1 mm/s, a3 = -4.05 mm/s, b3 = -17.83 mm/s, w = 6.16 1/s.

The secondary flows are defined by adding a time-dependent scaling factor to the axial W_{max} function, as shown in Equations (4) and (5).

$$Velocity\ in\ RL\ direction = percRL(t) * W_{max}(t) \tag{4}$$

$$Velocity\ in\ AP\ direction = percAP(t) * W_{max}(t) \tag{5}$$

The scaling functions are calculated by fitting a Fourier series in a way to discrete data points defined by the percentage amounts of secondary flow in right-to-left (percRL) and anterior-to-posterior (percAP) direction from the healthy H-25 case, see Equations (6) and (7), respectively.

$$\begin{aligned} percRL(t) = \; & a0 + a1 \cdot cos(tw) + b1 \cdot sin(tw) + a2 \cdot cos(2tw) + b2 \cdot sin(2tw) \\ & + a3 \cdot cos(3tw) + b3 \cdot sin(3tw) + a4 \cdot cos(4tw) + b4 \cdot sin(4tw) \\ & + a5 \cdot cos(5tw) + b5 \cdot sin(5tw) \end{aligned} \tag{6}$$

With the coefficients a0 = 0.4299, a1 = 0.02858, b1 = -0.01651, a2 = -0.06968, b2 = 0.1822, a3 = 0.08118, b3 = -0.03334, a4 = -0.04685, b4 = 0.03559, a5 = 0.01111, b5 = 0.007443, w = 6.175 1/s.

$$\begin{aligned} percAP(t) = {} & a0 + a1 \cdot cos(tw) + b1 \cdot sin(tw) + a2 \cdot cos(2tw) + b2 \cdot sin(2tw) \\ & + a3 \cdot cos(3tw) + b3 \cdot sin(3tw) + a4 \cdot cos(4tw) + b4 \cdot sin(4tw) \\ & + a5 \cdot cos(5tw) + b5 \cdot sin(5tw) + a6 \cdot cos(6tw) + b6 \cdot sin(6tw) \\ & + a7 \cdot cos(7tw) + b7 \cdot sin(7tw) + a8 \cdot cos(8tw) + b8 \cdot sin(8tw) \end{aligned} \tag{7}$$

With the coefficients a0 = -0.1456, a1 = 0.1055, b1 = -0.03278, a2 = -0.004963, b2 = -0.1124, a3 = -0.002429, b3 = 0.01333, a4 = -0.006754, b4 = -0.02793, a5 = 0.01831, b5 = 0.006278, a6 = 0.0004706, b6 = -0.02768, a7 = 0.002484, b7 = 0.006463, a8 = 0.006328, b8 = -0.00955, w = 6.16 1/s.

All Fourier series fittings were carried out with 95 % confidence bounds and $R^2 > 0.96$.
