# Supplementary material for: Investigation of hemodynamic bulk flow patterns caused by aortic stenosis using a combined 4D Flow MRI-CFD framework
Source: PLoS Comput Biol. 2025 Mar 27;21(3):e1012467. doi: 10.1371/journal.pcbi.1012467 (PMC11996075; doi:10.1371/journal.pcbi.1012467)
Supplement: S4 Methods — (PDF) [file pcbi.1012467.s005.pdf]

S4 Method. Mathematical derivation and explanation of all hemodynamic parameters of interest quantified in the study.

Viscous Shear Stress

Viscous shear stresses (VSS) are caused by molecular diffusion and are present for both laminar and turbulent flow. They are responsible for the strain rate which leads to the deformation of fluid elements. Viscous stresses can be written in the form of a stress tensor:

$$T = \begin{pmatrix} 2\mu\frac{\partial u}{\partial x} & \mu(\frac{\partial v}{\partial x}+\frac{\partial u}{\partial y}) & \mu(\frac{\partial w}{\partial x}+\frac{\partial u}{\partial z}) \\ " & 2\mu\frac{\partial v}{\partial y} & \mu(\frac{\partial w}{\partial y}+\frac{\partial v}{\partial z}) \\ " & " & 2\mu\frac{\partial w}{\partial z} \end{pmatrix} = \begin{pmatrix} \sigma_{xx} & \tau_{xy} & \tau_{xz} \\ \tau_{yx} & \sigma_{yy} & \tau_{yz} \\ \tau_{zx} & \tau_{zy} & \sigma_{zz} \end{pmatrix} \tag{1}$$

where the diagonal entries σ represent the normal stresses acting onto the fluid elements and the remaining elements τ describe the shear stresses. Here, μ is the fluid dynamic viscosity and the variables u, v, and w describe the velocity components in x, y and z-direction, respectively, in laminar flows or their statistically averaged quantities in turbulent flows. Utilizing the index notation, the elements of the viscous stress tensor are rewritten as follows:

$$\tau_{ij,viscous} = \mu\left(\frac{\partial u_i}{\partial x_j}+\frac{\partial u_j}{\partial x_i}\right) = 2\mu D_{ij} \tag{2}$$

D_{ij} is the strain rate tensor and u_i defines the i-component of the velocity in laminar flows or its statistically averaged quantity in turbulent flows. Often, a simplified scalar representative stress is advantageous for further analysis, especially in numerical applications, such as the prediction of RBC damage. For viscous stress, the following formulation, among other equivalent expressions, represents the three-dimensional stress conditions [1]:

$$\tau_{viscous} = \frac{1}{\sqrt{3}}\sqrt{\sigma_{xx}{}^2+\sigma_{yy}{}^2+\sigma_{zz}{}^2-(\sigma_{xx}\sigma_{yy}+\sigma_{xx}\sigma_{zz}+\sigma_{yy}\sigma_{zz})+3(\tau_{xy}{}^2+\tau_{xz}{}^2+\tau_{yz}{}^2)} \tag{3}$$

Reynolds Shear Stress

In an in-vitro study by Gülan et al. [2], a positive correlation was identified between the total flow stress on a fluid particle, i.e., viscous stresses and Reynolds stresses, and the severity of vessel diameter reduction in an aortic phantom. Thus, these stresses might serve as a parameter to localize regions of higher impact onto the RBCs. [2] When the Boussinesq's assumption is employed, Equation (4) is obtained [1, 3]:

$$\tau_{ij,Reynolds} = -\rho\overline{u'_i u'_j} = \mu_t\left(\frac{\partial u_i}{\partial x_j} + \frac{\partial u_j}{\partial x_i}\right) - \frac{2}{3}\rho k \delta_{ij} \tag{4}$$

with μ_t being the eddy viscosity, ρ the density, k the turbulence kinetic energy and δ_{ij} the Kronecker delta. The eddy viscosity μ_t is calculated depending on the chosen turbulence model, e.g., determined by the turbulent kinetic energy k and the turbulent frequency ω for the SST model.

Total Shear Stress

In conclusion, the definition of the total shear stress (TSS) tensor elements in index notation is:

$$\begin{aligned}\tau_{ij,total} = \tau_{ij,viscous} + \tau_{ij,Reynolds} &= \mu\left(\frac{\partial u_i}{\partial x_j} + \frac{\partial u_j}{\partial x_i}\right) + \mu_t\left(\frac{\partial u_i}{\partial x_j} + \frac{\partial u_j}{\partial x_i}\right) - \frac{2}{3}\rho k \delta_{ij} \\ &= (\mu + \mu_t)\left(\frac{\partial u_i}{\partial x_j} + \frac{\partial u_j}{\partial x_i}\right) - \frac{2}{3}\rho k \delta_{ij}\end{aligned} \tag{5}$$

The corresponding scalar equivalent stress ultimately equals to Equation (6):

$$\begin{aligned}\tau_{total} &= \frac{1}{\sqrt{3}} \cdot \; \dots \\ &\dots \sqrt{\sigma_{xx,t}{}^2 + \sigma_{yy,t}{}^2 + \sigma_{zz,t}{}^2 - (\sigma_{xx,t}\sigma_{yy,t} + \sigma_{xx,t}\sigma_{zz,t} + \sigma_{yy,t}\sigma_{zz)} + 3(\tau_{xy,t}{}^2 + \tau_{xz,t}{}^2 + \tau_{yz,t}{}^2)}\end{aligned} \tag{6}$$

Where $\sigma_{ij,t}$ is the abbreviation of the total stress component $\sigma_{ij,\mathrm{total}}$.

Wall Shear Stress

In the cardiovascular context, the wall shear stress (WSS) is defined as the friction stress of the blood flow applied tangentially onto the vessel wall. WSS can be calculated by multiplying the blood viscosity with the local velocity gradient, or the shear rate at the wall. WSS is a trigger for vessel wall remodeling, as it has an impact on the endothelial cell function. Therefore, abnormalities and increasing flow rates can lead

to the development of diseases such as atherosclerosis or aneurysms. Therefore, it often serves as a marker for abnormal blood flow and the precise measurement is of particular clinical interest [4].

Turbulent Kinetic Energy

The turbulent kinetic energy (TKE) can be calculated from the Reynolds normal stresses and serves as a characteristic scale for the extent of turbulence by evaluating the kinetic energy carried by turbulent fluctuations $u_i{}'$:

$$TKE = \frac{1}{2}\overline{u'_\iota u'_\iota} = \frac{1}{2}\overline{((u')^2 + (v')^2 + (w')^2)} \tag{7}$$

According to Itatani et al. [5], TKE is especially suitable for analysis of pathological turbulent blood flows that are strongly disturbed, as this quantity is independent from the in-vivo measurement resolution. They further empathize its use for AS flow hemodynamics characterized by a high velocity flow jet. [5]

Helicity

3D flow is characterized by a volumetric swirling behavior including helical spiraling. This torsion is described by helicity H, the scalar product of the space- and time-dependent velocity $\boldsymbol{V}(\boldsymbol{x};t)$ and vorticity field $\omega(\boldsymbol{x};t) = \nabla \times \boldsymbol{V}(\boldsymbol{x};t)$:

$$H(\boldsymbol{x},t) = \boldsymbol{V}(\boldsymbol{x};t) \cdot \omega(\boldsymbol{x};t) \tag{8}$$

High helicity is particularly present in blood flow fields altered by aortic valve diseases and therefore frequently investigated for cardiovascular diseases and the efficiency of the flow behavior. It indicates high cross-linked characteristics of vortex filaments which lead to local peaks of mechanical stress. [5]

A corresponding normalized quantity is introduced by Morbiducci et al. [6], the local normalized helicity LNH:

$$LNH(\boldsymbol{x};t) = \frac{\boldsymbol{V}(\boldsymbol{x};t) \cdot \omega(\boldsymbol{x};t)}{|\boldsymbol{V}(\boldsymbol{x};t)||\omega(\boldsymbol{x};t)|}, -1 \leq LNH \leq 1 \tag{9}$$

References

1. Faghih MM, Sharp MK. Modeling and prediction of flow-induced hemolysis: a review. Biomech Model Mechanobiol. 2019;18:845–81. doi:10.1007/s10237-019-01137-1.
2. Gülan U, Lüthi B, Holzner M, Liberzon A, Tsinober A, Kinzelbach W. An in vitro investigation of the influence of stenosis severity on the flow in the ascending aorta. Med Eng Phys. 2014;36:1147–55. doi:10.1016/j.medengphy.2014.06.018.
3. Konnigk L, Torner B, Bruschewski M, Grundmann S, Wurm F-H. Equivalent Scalar Stress Formulation Taking into Account Non-Resolved Turbulent Scales. Cardiovasc Eng Technol. 2021;12:251–72. doi:10.1007/s13239-021-00526-x.
4. Ha H, Lantz J, Ziegler M, Casas B, Karlsson M, Dyverfeldt P, Ebbers T. Estimating the irreversible pressure drop across a stenosis by quantifying turbulence production using 4D Flow MRI. Sci Rep. 2017;7:46618. doi:10.1038/srep46618.
5. Itatani K, Sekine T, Yamagishi M, Maeda Y, Higashitani N, Miyazaki S, et al. Hemodynamic Parameters for Cardiovascular System in 4D Flow MRI: Mathematical Definition and Clinical Applications. Magn Reson Med Sci. 2022;21:380–99. doi:10.2463/mrms.rev.2021-0097.
6. Morbiducci U, Ponzini R, Rizzo G, Cadioli M, Esposito A, Cobelli F de, et al. In vivo quantification of helical blood flow in human aorta by time-resolved three-dimensional cine phase contrast magnetic resonance imaging. Ann Biomed Eng. 2009;37:516–31. doi:10.1007/s10439-008-9609-6.
