# Supplementary figures and images for: Investigation of hemodynamic bulk flow patterns caused by aortic stenosis using a combined 4D Flow MRI-CFD framework
Source: PLoS Comput Biol. 2025 Mar 27;21(3):e1012467. doi: 10.1371/journal.pcbi.1012467 (PMC11996075; doi:10.1371/journal.pcbi.1012467)

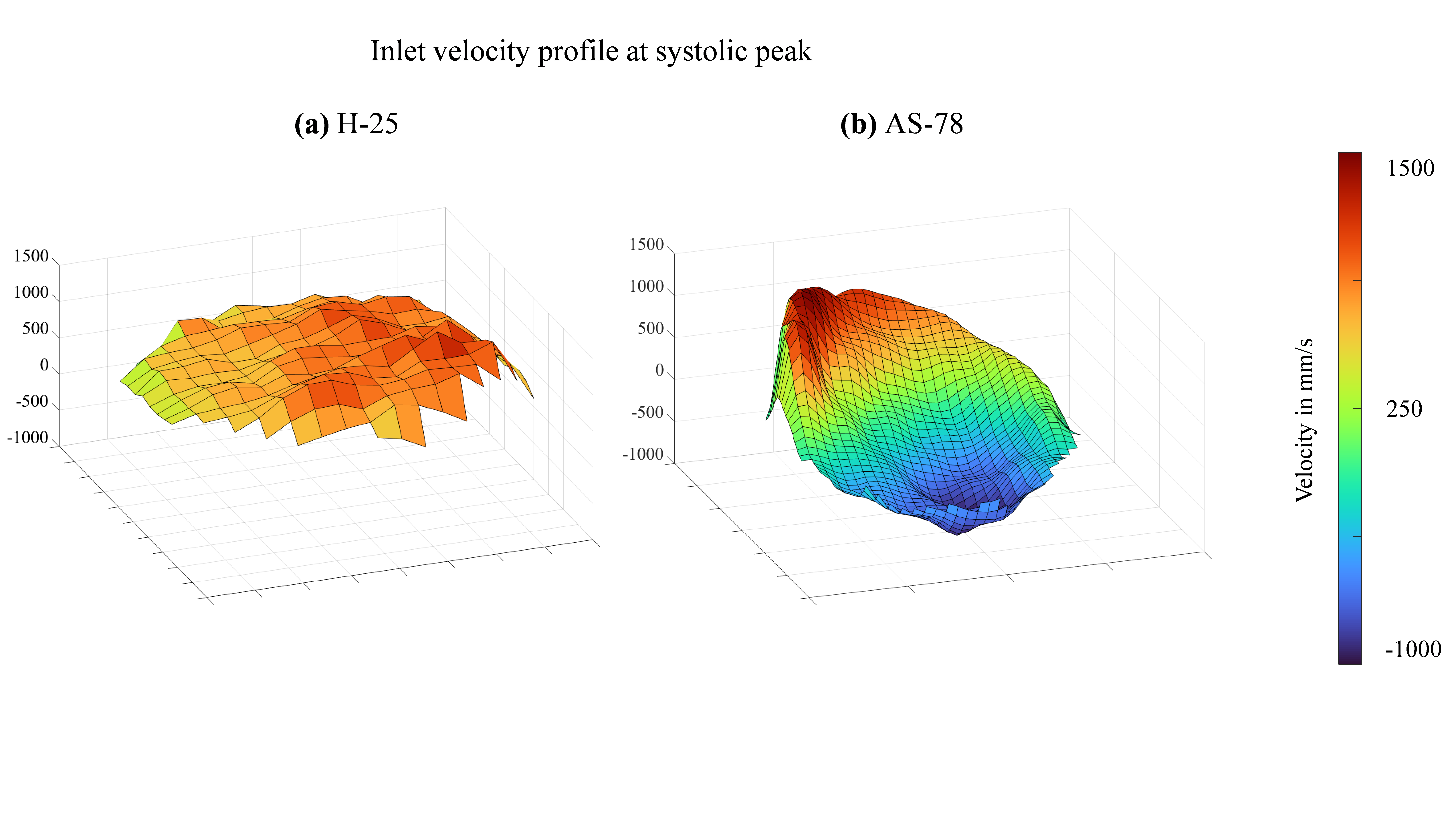

Supplement: S1 Fig — Prescribed inlet velocity profiles at systolic peak for (a) H-25 and (b) AS-78. The inlet boundary conditions of the H-25 and AS-78 case were defined by 4D Flow MRI-derived velocity profiles. The respective profiles at systolic peak are shown in S3 Fig. The profile for the healthy case shows the typical nearly parabolic profile, whereas the AS profile is characterized by a strong excentric velocity peak, even leading to a small portion of backflow. (TIF) [file pcbi.1012467.s006.tif]

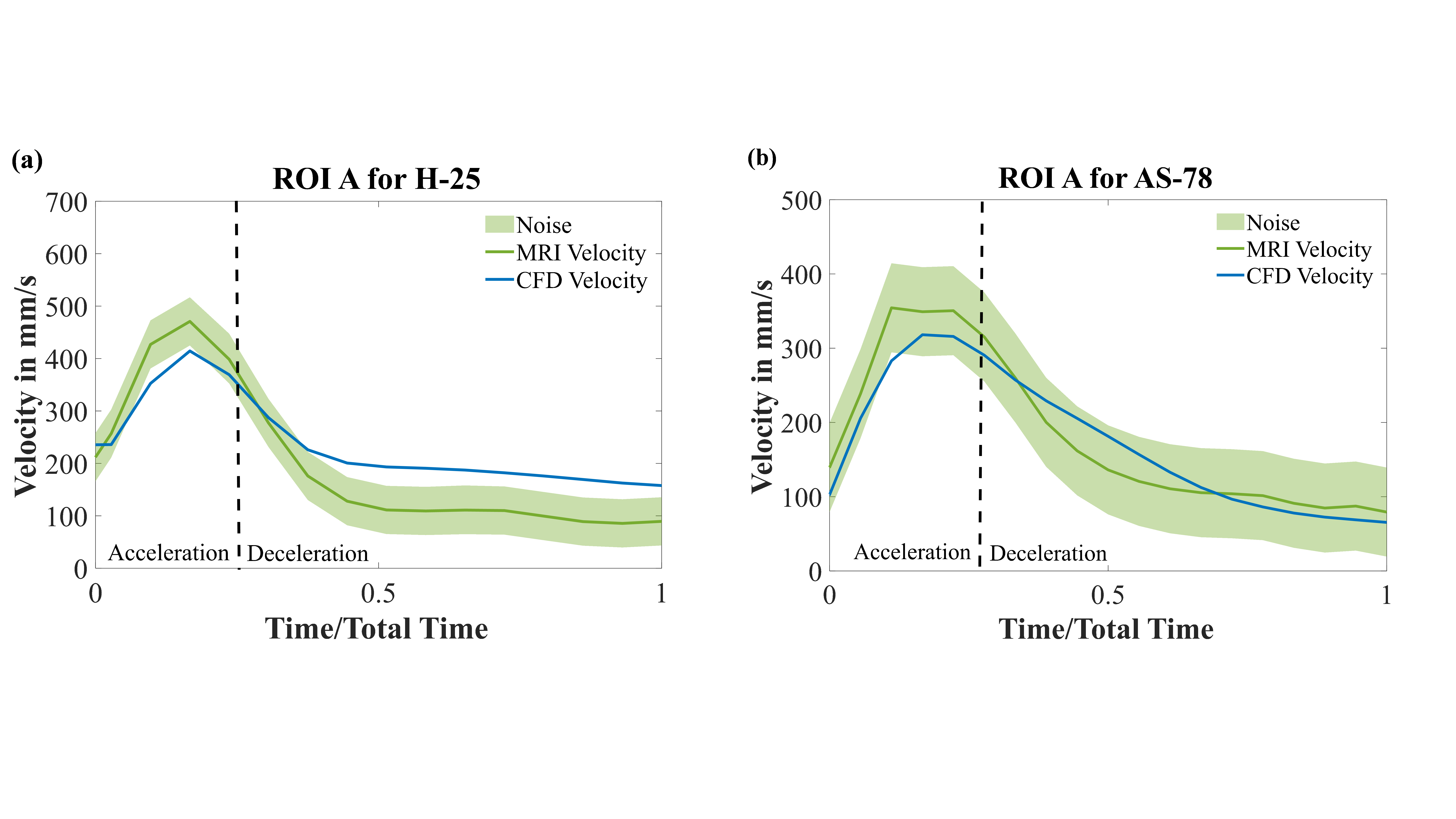

Supplement: S2 Fig — Validation results of the 4D Flow MRI-based model, each plot compares the 4D Flow MRI measured values with the corresponding numerically determined velocities. (a) Area-averaged temporal evolution of velocity for H-25 case on ROI A. (b) temporal evolution of velocity for AS-78 case on ROI A. (TIF) [file pcbi.1012467.s007.tif]
