# Supplementary material for: Investigation of hemodynamic bulk flow patterns caused by aortic stenosis using a combined 4D Flow MRI-CFD framework
Source: PLoS Comput Biol. 2025 Mar 27;21(3):e1012467. doi: 10.1371/journal.pcbi.1012467 (PMC11996075; doi:10.1371/journal.pcbi.1012467)
Supplement: S1 Result — The velocity contour maps at systolic peak are shown for ROIs A, B and C for both subject cases. The large difference between low-resolution 4D Flow MRI scans and high-resolution CFD simulation can be observed. (PDF) [file pcbi.1012467.s008.pdf]

S1 Result. 4D Flow MRI and CFD velocity contour maps on different cross-sectional planes for H-25 and AS-78.

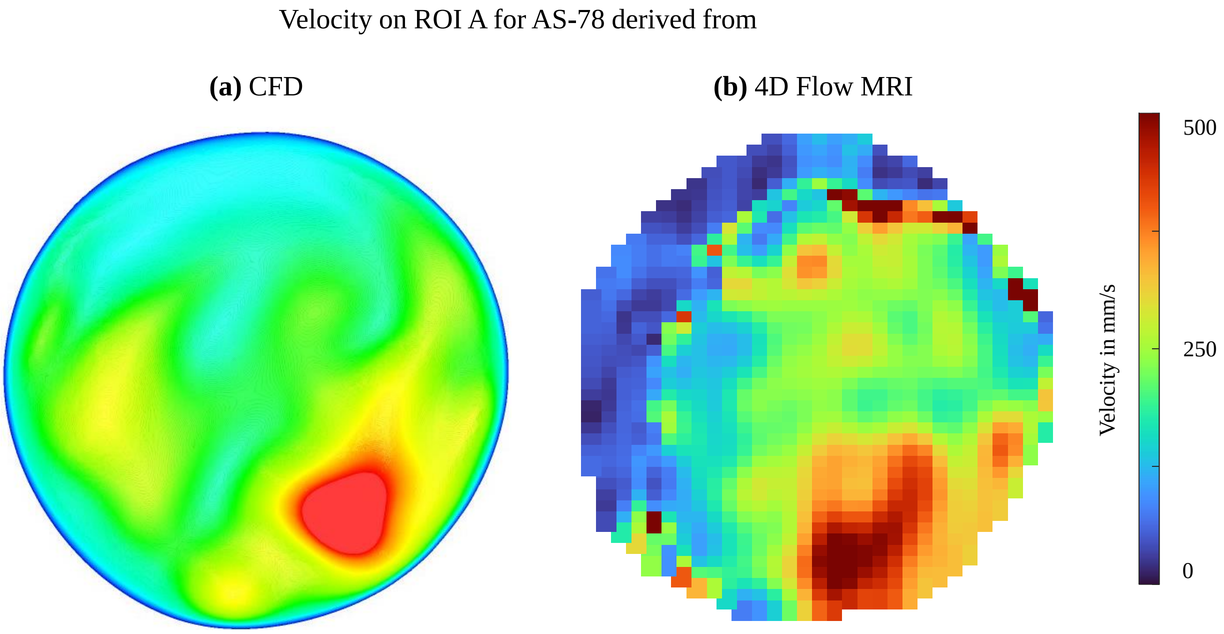

Fig A in S1 Result in Velocity maps for AS-78 on ROI A during systole. Left: high-resolution CFD velocity contour. Right: low-resolution 4D Flow MRI velocity contour

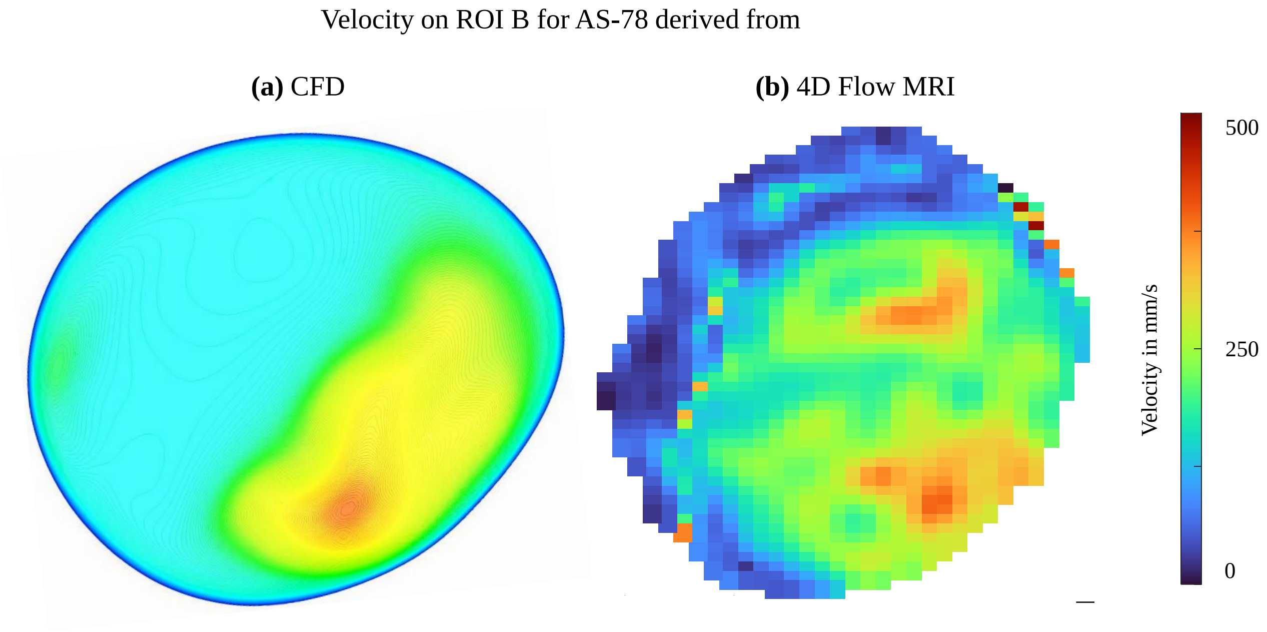

Fig B in S1 Result Velocity maps for AS-78 on ROI B during systole. Left: high-resolution CFD velocity contour. Right: low-resolution 4D Flow MRI velocity contour

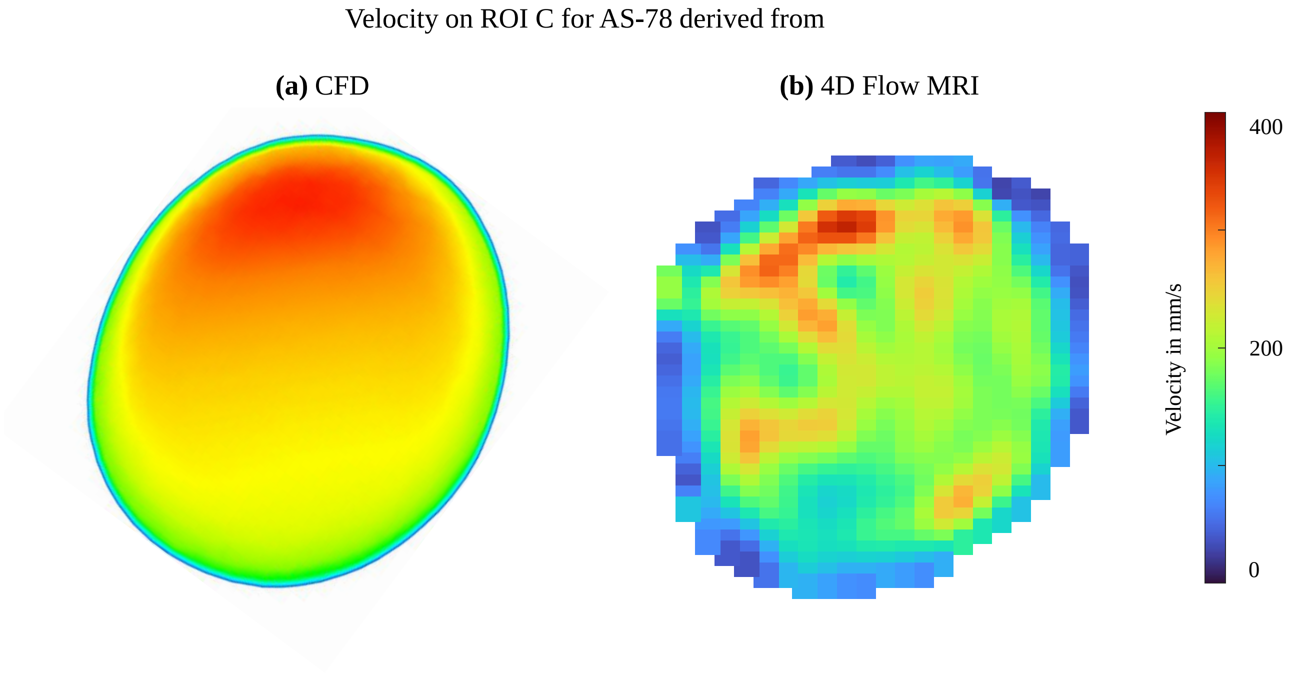

Fig C in S1 Result Velocity maps for AS-78 on ROI C during systole. Left: high-resolution CFD velocity contour. Right: low-resolution 4D Flow MRI velocity contour

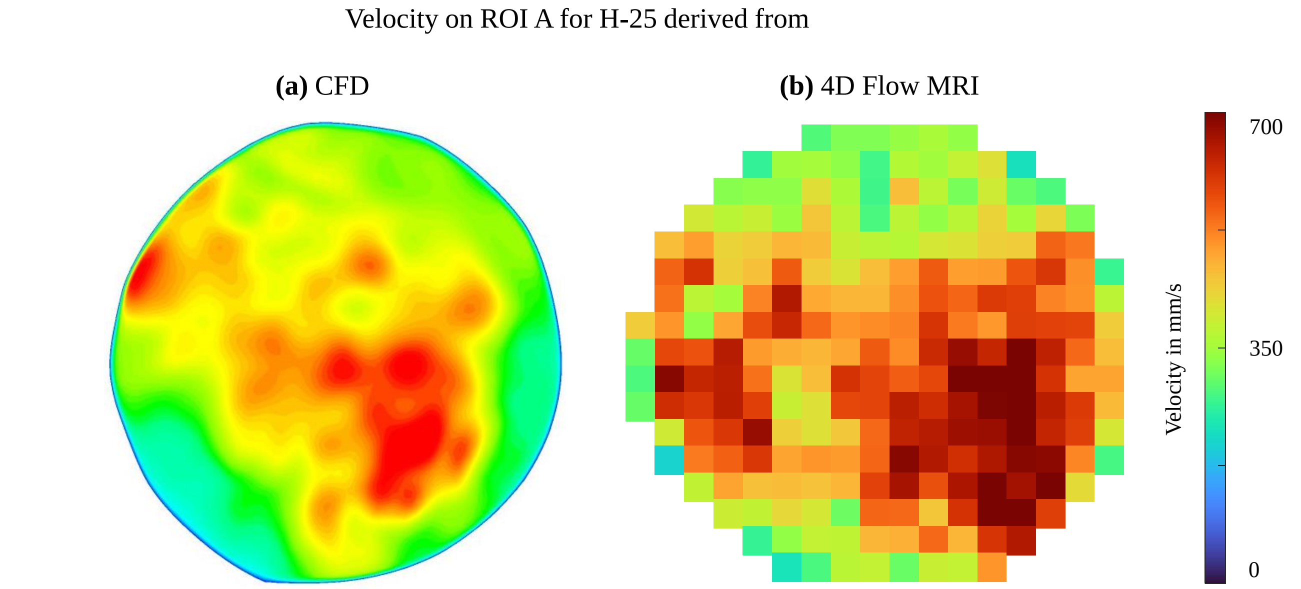

Fig D in S1 Result Velocity maps for H-25 on ROI A during systole. Left: high-resolution CFD velocity contour. Right: low-resolution 4D Flow MRI velocity contour

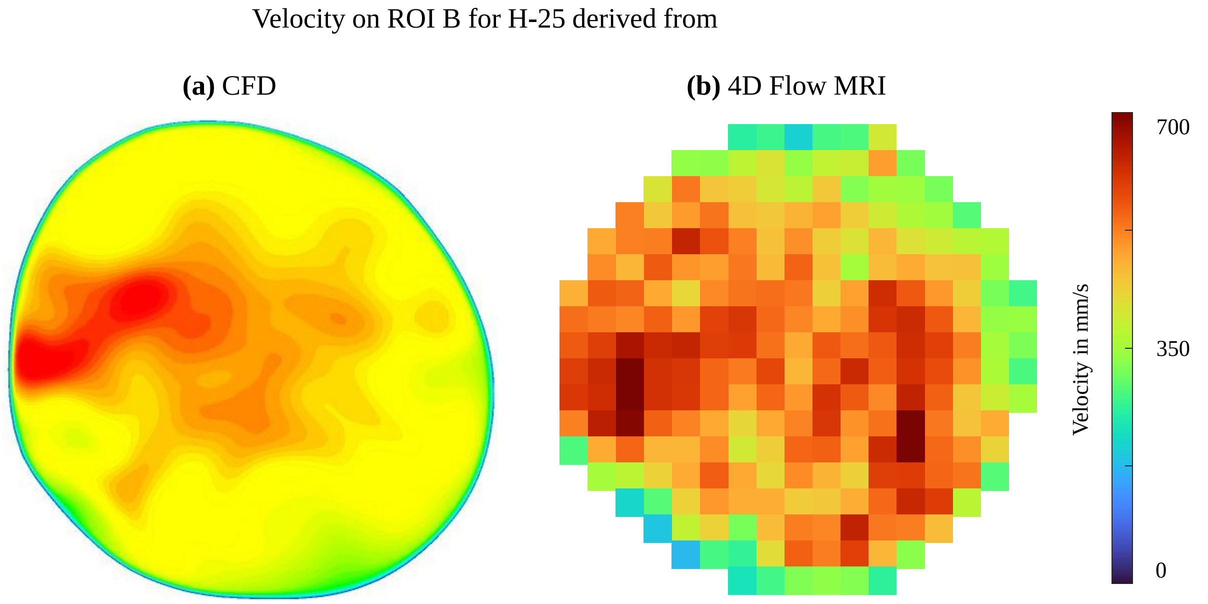

Fig E in S1 Result Velocity maps for H-25 on ROI B during systole. Left: high-resolution CFD velocity contour. Right: low-resolution 4D Flow MRI velocity contour

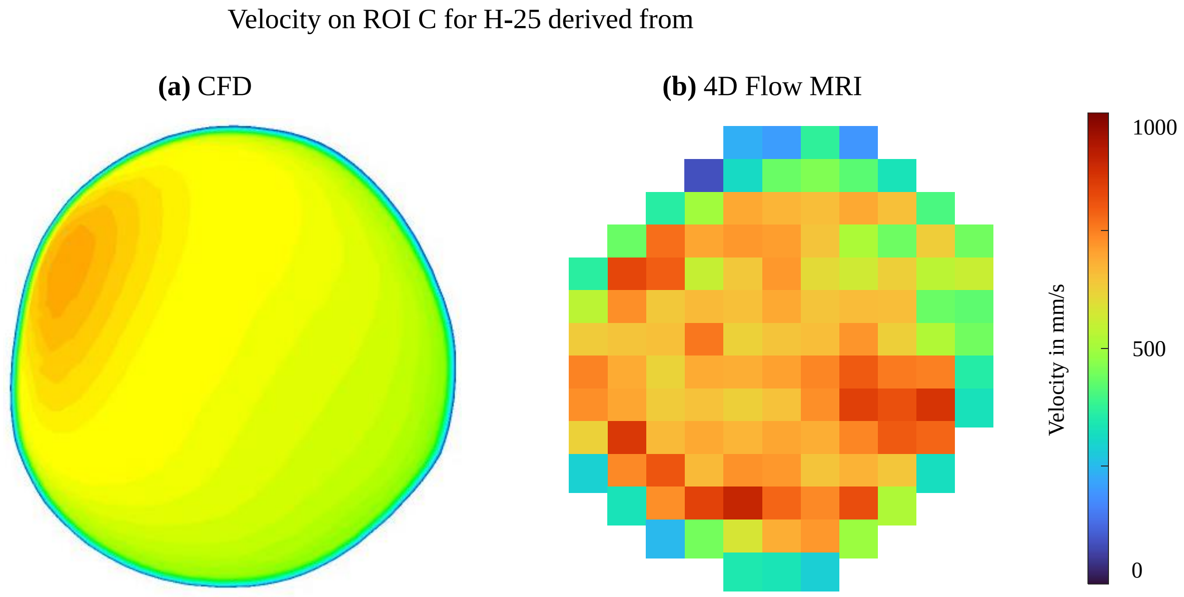

Fig F in S1 Result Velocity maps for H-25 on ROI C during early systole. Left: high-resolution CFD velocity contour. Right: low-resolution 4D Flow MRI velocity contour
